# Cost-Effective Components of a Patient-Reported Symptom Monitoring System for Chemotherapy

**DOI:** 10.1001/jamanetworkopen.2025.42289

**Published:** 2025-11-10

**Authors:** Kathi Mooney, Minkyoung Yoo, Elizabeth S. Sloss, Bridget Nicholson, Natalya S. Alekhina, Eli Iacob, Richard Nelson

**Affiliations:** 1 University of Utah College of Nursing, Salt Lake City; 2 Department of Internal Medicine, University of Utah School of Medicine, Salt Lake City

## Abstract

**Importance:**

Electronic patient-reported outcome (ePRO) systems often combine a variety of components to manage and improve cancer symptom burden. While shown to reduce symptom severity, there are few studies that have assessed whether the included components are cost-effective.

**Objective:**

To evaluate which components or combinations of components of Symptom Care at Home (SCH), an ePRO for adults with cancer receiving treatment, were most cost-effective.

**Design, Setting, and Participants:**

This 5-group economic evaluation used data from a randomized clinical trial conducted from 2017 to 2020 across 2 cancer centers located in Utah and Georgia. Participants included adults with cancer starting a chemotherapy protocol. Data were analyzed from 2021 to 2024.

**Interventions:**

Participants were randomized into 1 of 5 groups receiving different combinations of the SCH intervention components, including automated self-management coaching and/or nurse practitioner (NP) follow-up. All groups completed daily ePRO symptom reporting across 11 symptoms and received at least 1 SCH component.

**Main Outcomes and Measures:**

Cost-effectiveness was assessed using Markov simulation models and incremental cost-effectiveness ratios (ICERs) incorporating symptom burden, unplanned hospitalizations and emergency department visits, and SCH development and operational costs by group. Symptom burden was calculated as the summed symptom severity scores over the study period.

**Results:**

Among 757 adult participants (mean [SD] age, 59.2 [12.9] years; 463 [61.2%] female), 474 (62.6%) were married or partnered; 240 participants (31.7%) self-identified as Black, 29 participants (3.8%) as Hispanic, and 488 participants (64.5%) as White. In the base case analysis, 6-month total costs varied from $14 590 (NP follow-up with decision support) to $23 992 (NP follow-up only). The complete SCH intervention had the highest cost-effectiveness. The ICER for the complete SCH vs NP-only group was $4957. The complete SCH intervention had the highest probability of being cost-effective, at nearly all positive willingness-to-pay values, including very low thresholds.

**Conclusions and Relevance:**

This cost-effectiveness study of ePRO symptom management components found that the complete SCH intervention with all component parts was the most cost-effective treatment strategy. From a value-driven perspective, comprehensive ePRO systems both improve quality through reduced symptom burden and were most cost-effective.

## Key Points

**Question:**

Which component or combination of components of an electronic patient-reported outcome (ePRO) system are most cost-effective for managing symptom burden in patients receiving chemotherapy?

**Findings:**

In this economic evaluation including 757 participants randomized to 1 of 5 combinations of ePRO strategies, the complete ePRO intervention, including self-management coaching and nurse practitioner follow-up for severe symptoms, had the highest cost-effectiveness, while the group with self-management coaching plus an activity tracker had the lowest.

**Meaning:**

These findings suggest that comprehensive ePRO systems that integrate multiple intervention components offer the greatest value in terms of symptom control and cost-effectiveness.

## Teaser

This economic evaluation study evaluate which components or combinations of components of an electronic patient-reported outcomes system for adults with cancer receiving treatment was most cost-effective.

## Introduction

In the continuously evolving health care landscape, innovative digital symptom monitoring and management interventions have been shown to transform patient experience and reduce symptom burden and unplanned health care utilization. These automated digital tools systematically collect electronic patient-reported outcomes (ePROs). Based on the ePRO reports, interventions to reduce symptom burden and improve quality of life are offered. Interventions to decrease symptom burden can be automated, delivered over the phone, or provided at an in-person clinic visit. They can also have several variations of intervention components, which may be tailored to the reported symptom severity. Both academically developed and commercially available ePRO systems vary in the components they offer, their development costs, the degree of predeployment testing and optimization, as well as the cost to maintain and operate the systems.

Significant evidence supports the use of ePRO systems to improve patient symptom burden and decrease acute care utilization during cancer treatment.^1,2,3^ Given these benefits, focus has now shifted to broad implementation in cancer care.^4,5,6,7^ While effective clinical pathways exist to support the adoption of these systems and engage both patients and clinicians, academic and community oncology practices must also consider the cost-effectiveness of systems in relationship to the desired outcomes. However, there are few economic analyses of these systems and what combination of intervention components are most cost-effective for each system.

Over time, we have sequentially developed and tested an ePRO-based cancer symptom monitoring and management system, Symptom Care at Home (SCH), that has well-established efficacy.^2,8^ SCH was designed to support daily remote symptom monitoring, provide automated and tailored self-management coaching, and initiate a nurse practitioner (NP) telephone follow-up for symptoms rated 4 or higher on a 0 to 10 severity scale.

In a prior randomized trial by Mooney et al,^2^ 280 participants were randomized to SCH or enhanced usual care (UC), with both groups reporting daily severity of 11 symptoms via the automated system. Only SCH participants received automated self-management coaching and NP follow-up for poorly controlled symptoms. UC participants received standard symptom management from their oncology team. Compared with UC, SCH participants reported significantly lower overall symptom severity (mean reduction, 3.59 points; *P* < .001), representing 43% lower symptom burden than UC. They also experienced 67% fewer severe-rated symptom days (*P* < .001) and 39% fewer moderate severity days (*P* < .001). All individual symptoms except diarrhea also showed significant improvement in the SCH group, demonstrating the clinically meaningful efficacy of the SCH intervention.^2^

To refine SCH for broader implementation, we conducted a prospective component evaluation to assess whether all elements of the SCH intervention were necessary to achieve optimal symptom control.^9^ We found that the full SCH model led to the lowest symptom burden across all subcomponent groups. The published findings provide additional details on the design, sample characteristics, and symptom burden outcomes.^9^ In this study, we extend that work by evaluating the cost-effectiveness of the SCH intervention components to determine which components are most cost-effective in finalizing the intervention prior to implementation in oncology practice.

## Methods

This economic evaluation was conducted as part of a randomized clinical trial reviewed and approved by institutional review boards from each participating organization. All participants provided written informed consent prior to enrollment. This study was conducted in accordance with the Consolidated Health Economic Evaluation Reporting Standards (CHEERS) reporting guideline.

### Design

We conducted a multisite 5-group prospective randomized clinical trial study in cancer centers located in Utah and Georgia. We did not use a UC control group due to the efficacy demonstrated by SCH in a previous study,^8^ as there was no longer equipoise for a control condition. Participants were compensated $150 divided into 2 payments. The study was registered in ClinicalTrials.gov (NCT0277925).

The primary aim of our randomized clinical trial was to determine which component or combination of components from the SCH intervention provided the greatest reduction in symptom. Here, we report the prespecified secondary aim: to determine which component or combination of components was most cost-effective, as the prespecified secondary aim.

### Participant Eligibility and Recruitment Sites

We recruited participants from the medical oncology practices at Huntsman Cancer Institute in Salt Lake City, Utah, and Emory University Winship Cancer Institute, including Grady Hospital, in Atlanta, Georgia. Recruitment occurred from August 7, 2017, through January 17, 2020. Patients were eligible to enroll if they were aged at least 18 years, had a cancer diagnosis, had a life expectancy of at least 3 months, and spoke English. They needed to be starting a chemotherapy course planned for 3 or more cycles. They were required to have daily access and ability to use a telephone, although it did not need to be a smart phone, as SCH is an interactive voice response system that transmits data using the traditional telephone network. Patients were excluded if they were also to receive concurrent radiation therapy, since they would have daily contact with oncology clinicians for symptom concerns.

### Randomization

Consenting participants were randomly assigned to 1 of the 5 treatment groups using the REDCap software (Vanderbilt). Randomization was independently generated by site in blocks of 10, with 2 to 3 participants per block, and stratified by sex. Once randomized, staff collected baseline data and oriented the participant to their treatment group. Participants self-reported demographic characteristics, including race and ethnicity (including, Black, Hispanic, and White) and sex.

### Intervention

All groups used the SCH system to report daily whether any of 11 common treatment-related symptoms were present and if present, the severity on a scale of 1 to 10, with 10 indicating most severe. The components received depended on intervention group assignment.

The 5 intervention groups were (1) automated self-management coaching alone (SCC; 143 participants), (2) automated self-management coaching with an activity tracker visible to the participant (SCC-AT; 144 participants), (3) NP follow-up using best practices for symptom management (NP-only; 148 participants); (4) NP plus guideline-based clinical decision support for symptom management (NP-DSS; 155 participants), and (5) the complete SCH intervention that included automated self-management coaching and NP follow-up with decision support (167 participants).

### Cost-Effectiveness Analysis

The purpose of this study was to perform a cost-effectiveness analysis to determine what combination of the SCH intervention components is most cost-effective. Our outcome metric was the incremental cost-effectiveness ratio (ICER), which measures the additional cost required to gain 1 additional unit of effectiveness. This study was conducted from a health care sector perspective, incorporating both payer-covered costs and patient out-of-pocket expenditures.

### Model Structure

**Figure 1.**
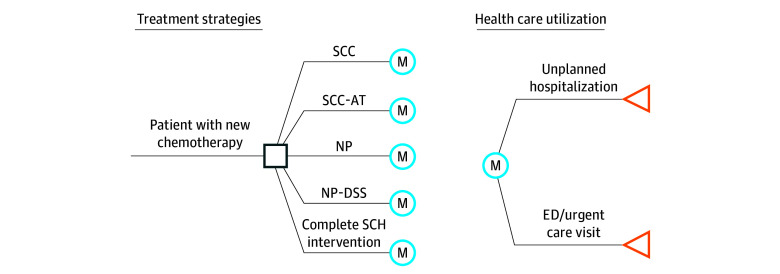
Illustration of the Structure of the Markov Model of Treatment Groups and Unplanned Health Care Utilization ED indicates emergency department; SCC, the group that received automated self-management coaching (SCC) alone; SCC-AT, SCC with an activity tracker visible to the participant; NP, nurse practitioner (NP) follow-up using best practices for symptom management; NP-DSS, NP follow-up plus guideline-based clinical decision support for symptom management (DSS); and complete SCH, participants received SCC and activity tracker with NP follow-up with DSS.

A Markov simulation model was constructed to estimate the cost-effectiveness of the 5 intervention groups. The model is designed to simulate progression through health care utilization outcomes, focusing events such as unplanned hospitalizations and emergency department (ED) visits. The model was run using a 1-week cycle over a 26-week time (or 6-month) horizon on a modeled population of 10 000 hypothetical patients. A 26-week time horizon was selected based on the maximum participation duration, regardless of the patient’s treatment schedule. Each intervention group was evaluated with respect to both effectiveness and cost. No mortality was observed; hence, it was not considered in the model. Unplanned health care utilization was collected from the medical record. The structure of the Markov model is illustrated in Figure 1. The model was programmed in TreeAge Pro 2018 software (TreeAge Software).

### Effectiveness

The effectiveness of each group was measured by a 1-point reduction in total symptom burden score, which ranges from 0 to 10, with 0 indicating the most effective status and 10 representing the worst, defined using a weekly mean. The daily automated calls identified symptom presence, and if a symptom was present, a 1 to 10 scale was used to report severity and distress. Severity scores were categorized as 0, indicating symptom not endorsed; 1 to 3, mild; 4 to 7, moderate; and 8 to 10, severe. For each intervention group, the total burden was computed as the integral of the quadratic curve, with initial differences in group randomization adjusted formally through the analysis model. Details on the inferential analysis are discussed in elsewhere.^9^ Weekly symptom burden scores by group are presented in the eTable in Supplement 1.

### Cost

Our model included the costs of the interventions as well as costs of health care encounters that can be avoided with the intervention, such as unplanned hospitalizations and ED visits. The likelihood of unplanned hospitalization and ED visits was estimated by counting the number of each type of visit during the study’s follow-up period (Table 1). The mean number of ED visits, conditional on incurring any visits, the mean number of unplanned hospitalizations, conditional on incurring any visits, and the length of stay for unplanned hospitalization were also included.

**Table 1.** Input Parameters of Emergency Department Visit and Unplanned Hospitalization

Outcome	Mean (SD)				
	SCC	SCC-AT	NP	NP-DSS	Complete SCH
Patients, No.	143	147	150	157	171
Emergency department visit					
Weekly likelihood of having any visit, %	3.7 (7.9)	4.2 (10.8)	4.4 (9.8)	3.1 (6.6)	4.7 (14.4)
Visits for patients with ≥1 visit, No.	1.6 (0.8)	2.0 (1.8)	1.7 (1.0)	2.1 (2.9)	1.7 (1.8)
Unplanned hospitalization					
Weekly likelihood of having any visit ,%	3.4 (8.9)	2.5 (6.9)	3 (9.4)	2.0 (5.3)	2.7 (8.5)
Visits for patients with ≥1 visit, No.	1.3 (0.4)	1.3 (0.7)	1.4 (0.6)	1.7 (1.9)	1.3 (0.6)
LOS if any visit, d	4.7 (4.2)	5.0 (4.6)	5.2 (3.4)	3.6 (2.2)	4.7 (4.4)

Abbreviations: LOS, length of stay; NP, nurse practitioner follow-up using best practices for symptom management; NP-DSS, nurse practitioner follow-up plus guideline-based clinical decision support for symptom management; SCC, automated self-management coaching alone; SCC-AT, automated self-management coaching with an activity tracker visible to the participant; SCH, automated self-management coaching with activity tracker and nurse-practitioner follow-up with decision support; .

We used mean expenditure by event type from the Medical Expenditure Panel Survey^10^ to estimate costs for unplanned hospitalizations and ED visits. Weekly total estimated unplanned hospitalization costs were calculated as the product of the likelihood of hospitalization, the per-night hospitalization cost, and the mean length of stay. Similarly, weekly total ED costs were calculated by multiplying the likelihood of an ED visit by the mean cost per visit. All costs were adjusted to 2023 US dollars using the Personal Consumption Expenditures price index.^11^

SCH development costs included programming the SCH web interface, developing the SCH project script, testing and delivering production, and hosting and supporting fees for clinics, yielding a total value of $491 118 in 2023 US dollars. Additionally, the NP time spent on the intervention was reported at the start of the intervention. Mean monthly NP hours were 20 hours for NP-DSS and complete SCH groups, 11.5 hours for the NP-only group, and no NP hours for SCC and SCC-AT groups. The mean hourly wage for NPs in Utah^12^ was used to calculate total labor costs for the intervention. Total intervention cost per person for each group is summarized in Table 2.

**Table 2.** Input Parameters of Costs

Category	Cost, $^a^
SCH total development materials	491 118
SCH labor^b^	
Hourly wage for nurse practitioner in Utah	59.80
Monthly nurse practitioner time	
DSS groups, h	19.79
Non-DSS group, h	11.46
Intervention cost per patient	
SCC^c^	1253
NP^d^	1309
NP-DSS^e^	1343
Complete SCH intervention^f^	1340
SCC-AT^g^	1253
Health care	
Inpatient stay, per night, mean (SD)	3574 (122)
Emergency department visit, mean (SD)	1135 (33)

Abbreviations: SCH, Symptom Care at Home intervention, including self-management coaching with activity tracker and nurse-practitioner follow-up with decision support; NP, nurse practitioner follow-up using best practices for symptom management; NP-DSS, nurse practitioner follow-up plus guideline-based clinical decision support for symptom management; SCC, automated self-management coaching alone; SCC-AT, automated self-management coaching with an activity tracker visible to the participant.

^a^ All values reported as 2023 US dollars.

^b^ Source: Bureau of Labor Statistics,^12^ Bureau of Economic Analysis,^10^ Personal Consumption Expenditures 2023.^11^

^c^ Participants received SCC alone.

^d^ Participants received NP follow-up using best practices for symptom management. ^e^Participants received NP practitioner follow-up plus DSS.

^f^ Participants received complete SCH intervention, including SCC with AT and NP follow-up with DSS.

^g^ Participants received SCC with an AT visible to the participant.

### Statistical Analysis

The base case analysis used the point estimates (mean and SD) for each input parameter value. To determine model robustness regarding base case probabilities of unplanned health care use and cost estimates, we performed a probabilistic sensitivity analysis to assess the impact of uncertainty in all transition probabilities and costs (Table 3). The study was conducted using 1000 second-order Monte Carlo simulations for 1000 hypothetical patients, in which all model parameters varied simultaneously rather than individually, based on random drawings from a distribution. Transition probabilities were modeled using a β distribution, while cost estimates followed a γ distribution. Symptom burden scores were held constant across simulations, as we did not have individual-level data to estimate variability.

**Table 3.** Cost-Effectiveness Analysis Results

Strategy	Cost, 2023 $	Effectiveness^a^	ICER
**Base case**			
NP-DSS^b^	$14 590	5.3	0 [Reference]
Complete SCH^c^	$18 200	4.5	$4957
SCC-AT^d^	$18 796	6.9	Dominated^e^
SCC^f^	$21 283	6.5	Dominated^e^
NP^g^	$23 992	5.2	Dominated^e^
**Base case excluding development cost**			
NP-DSS^b^	$13 337	5.3	0 [Reference]
Complete SCH^c^	$16 947	4.5	$4957
SCC-AT^d^	$17 543	6.9	Dominated^e^
SCC^f^	$20 030	6.5	Dominated^e^
NP^g^	$22 739	5.2	Dominated^e^
**Probabilistic sensitivity analysis**			
NP-DSS^b^	$13 461	4.7	0 [Reference]
Complete SCH^c^	$18 654	5.5	$7130
SCC-AT^d^	$17 370	3.1	Dominated^e^
SCC^f^	$21 177	3.5	Dominated^e^
NP^g^	$24 487	4.8	Dominated^e^

Abbreviations: ICER, incremental cost-effectiveness ratio; NP, nurse practitioner follow-up using best practices for symptom management; NP-DSS, nurse practitioner follow-up plus guideline-based clinical decision support for symptom management; SCC, automated self-management coaching alone; SCC-AT, automated self-management coaching with an activity tracker visible to the participant; SCH, Symptom Care at Home intervention, including self-management coaching with activity tracker and nurse-practitioner follow-up with decision support.

^a^ Effectiveness measure was defined as the symptom burden score, with 0 indicating most effective status and 10, worst.

^b^ Participants received NP practitioner follow-up plus DSS.

^c^ Participants received complete SCH intervention, including SCC with AT and NP follow-up with DSS.

^d^ Participants received SCC with an AT visible to the participant.

^e^ Indicates the strategy was more costly and less effective than NP-DSS or complete SCH or less cost-effective than a combination of the 2 more efficient strategies.

^f^ Participants received SCC alone.

^g^ Participants received NP follow-up using best practices for symptom management.

All statistical analyses were performed using SAS software version 9.4 (SAS Institute). The cost-effectiveness model was developed and executed in TreeAge Pro Healthcare 2024 (TreeAge Software). Data were analyzed from 2021 to 2024.

## Results

### Demographics

Of 1244 patients found eligible and invited to participate, 884 (83.7%) consented to participate, with 757 participants (mean [SD] age, 59.2 [12.9] years; 463 [61.2%] female) reporting symptoms at least once and whose data were analyzed (127 participants did not participate after consenting: 18 became ineligible; 109 reconsidered and never participated after consent).^9^ There were no group differences between individuals who declined participation vs participants, but they were more likely to be Black, unmarried, or with less education.

There were 143 participants in the SCC group, 144 participants in SCC-AT; 148 participants in the NP-only group, 155 participants in the NP-DSS group, and 167 participants in the complete SCH group. The variation in group size was naturally occurring due to the complexity of randomization across the 5 groups in 2 accrual sites and did not significantly impact power.

Overall, 474 participants were married or partnered (62.6%), with 240 participants (31.7%) self-identifying as Black, 29 participants (3.8%) as Hispanic, and 488 participants (64.5%) as White. The most common cancer diagnoses were breast (132 participants [17.4%]), lung (107 participants [14.1%]), colorectal (99 participants [13.1%]), ovarian (63 participants [8.3%]), and pancreatic (62 participants [8.2%]). Nearly half of participants had metastatic cancer (369 participants [48.7%]). There were no significant differences across groups for patient demographic or disease characteristics. The mean (SD) time in the study was 69.6 (25.6) days, with a maximum of 26 weeks. The median (IQR) daily reporting adherence was 75.6% (3.9%-100%).

### Base Case

In our base case analysis, we found that the 6-month mean total cost, including intervention cost and unplanned health care costs, ranged from $14 590 (NP-DSS) to $23 992 (NP) (Table 3). The estimated 6-month symptom severity score was lowest for the complete SCH group, at 4.5, and highest for the SCC-AT group, at 6.9. The SCC, SCC-AT, and NP-only groups were eliminated from consideration because they were either absolutely dominated (more costly and less effective than NP-DSS or complete SCH) or extendedly dominated (less cost-effective than a combination of the 2 more efficient strategies). Among the 2 nondominated groups, the complete SCH group yielded greater effectiveness (ie, lower symptom burden score) compared with NP-DSS (5.3) but at higher costs, at $18 200 vs $14 590. The ICER for the complete SCH group compared with NP-DSS was $4957. In this study, the ICER indicates that the incremental cost of a 1-unit improvement of symptom burden was $4957.

### Sensitivity Analyses

**Figure 2.**
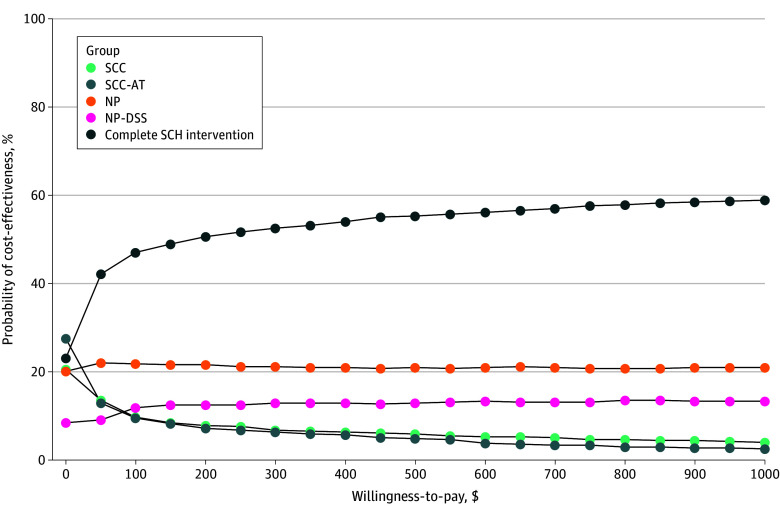
Cost-Effectiveness Acceptability Curve by Willingness-to-Pay Threshold SCC indicates automated self-management coaching (SCC) alone; SCC-AT, SCC with an activity tracker visible to the participant; NP, nurse practitioner (NP) follow-up using best practices for symptom management; NP-DSS, NP follow-up plus guideline-based clinical decision support for symptom management (DSS); and complete SCH, participants received SCC and activity tracker with NP follow-up with DSS.

The results from the probabilistic sensitivity analysis are consistent with those from the base case analysis (Figure 2). This figure is a cost-effectiveness acceptability curve that depicts the proportion of the 1000 Monte Carlo simulations in which each strategy is cost-effective for a range of willingness-to-pay (WTP) thresholds. For WTP thresholds more than approximately $20, the complete SCH intervention was the strategy that was most frequently cost-effective.

## Discussion

This cost-effectiveness study of the intervention components of the SCH ePRO monitoring and management system found that the most cost-effective component was the complete SCH intervention that included both automated self-care management coaching tailored to reported daily symptom severity paired with NP telephone follow-up for symptoms of moderate and higher severity using guideline-based symptom management decision support. The cost-effectiveness analysis considered development costs, operating costs, degree of symptom improvement, and unplanned health care utilization costs (ED visits and unplanned hospitalizations). These findings are consistent with our primary aim analysis of the symptom burden reduction of the various components that also favored the complete SCH system.^9^ This may reflect the favorable health care cost reduction that can be obtained when interventions are multicomponent, targeting different aspects of symptom care. In the case of SCH, the automated self-management coaching is tailored to the patient’s symptom report, providing just-in-time suggestions on what the patient should do. Symptoms that are poorly controlled and prone to escalating are addressed emergently by an NP. The combination provides both timely action for decreasing escalating symptoms and tailored patient guidance and reassurance in maintaining ongoing symptom control, resulting in reduced symptom burden, which results in cost-effectiveness.

The value of ePRO symptom monitoring combined with systematically delivered interventions is also multicomponent.^13^ Besides the demonstrated improvement in symptom control and reduction in unplanned health care utilization, these systems engage patients in their care and improve their care experience and feeling of being connected to the oncology team during the time between clinic visits.^14^ Value-driven care is a function of both quality and cost. Quality is a combined measure of care outcomes and patient experience. Components should demonstrate both quality and cost-effectiveness to optimize an ePRO monitoring and management system.

While overall there are few reports of cost-effectiveness, several groups have reported ePRO system cost-effectiveness compared with usual care.^6,15,16,17^ While variable in design and results, these evaluations demonstrate good value for the cost when the ePRO system was compared to standard symptom management. Further work is needed to fully understand the return on investment for oncology practices. One of the issues in cost-effectiveness studies is their application to actual clinical practice due to the restricted sample size of a study and the much larger number of patients that would benefit from an ePRO system in a practice setting over time. In addition, actual cost-effectiveness can vary based on the clinical context, capital investment, organizational structure, workflow processes, and the value assessment perspective taken for the analysis, whether patient, health care system, or societal.^18^

To our knowledge, there are limited, if any, publications other than ours that demonstrate optimizing the components of a digital ePRO system prior to moving forward with pragmatic implementation. As digital monitoring tools extend beyond assessment into the management of symptoms, it is key to understand, among the growing array of components that could be added, which ones actually add value These findings suggest that the optimal SCH intervention for improved symptom outcomes, positive patient experience, and cost-effectiveness requires our complete multicomponent SCH intervention.

### Limitations

While our systematic examination of the optimal SCH intervention components has numerous strengths, we also acknowledge several limitations. First the analysis focused solely on determining cost-effectiveness within components of SCH and was not designed to compare to current usual care. Understanding how ePRO symptom tools compare in cost-effectiveness to current symptom care practices or other ePRO systems would be useful when considering adoption among oncology practices. Additionally, in terms of our approach to the cost-effectiveness analysis, we did not use a standard utility measure, the quality-adjusted life-year, therefore limiting comparison to other cost-effectiveness studies. Since the primary intent of SCH is to decrease symptom burden, we used our rich longitudinal dataset of symptom severity rather than a quality-of-life measure that includes other factors that dilute the interpretation for symptom burden. We also only included Medical Expenditure Panel Survey data. which, as any data source, does have some limitations, such as indirect contributors, including lost wages or family caregiver costs in providing care. Additionally, we only included ED visits and unplanned hospitalization that occurred within the participating health systems. This may have underrepresented utilization, since use outside of the system would not have been counted. However, since the groups were randomized, we believe any underreporting would be equally distributed across groups.

## Conclusions

This economic evaluation found that the value of ePRO digital health symptom management components can be determined by examining components for quality and cost. We found that the complete SCH intervention was the most cost-effective intervention combination, demonstrating that ePRO interventions that are comprehensive and target several components of symptom care bring the greatest value.

The value of ePRO symptom monitoring and management for symptom control in oncology clinical practice is receiving increasing interest in both academic and community practices as well as in payer models, including the Centers for Medicare & Medicaid Services Enhancing Oncology Model program.^6,19,20^ Value-focused models incentivize the implementation of ePROs, yet reimbursement in fee-for-service models remain limited.^20^ Health system decisions to adopt these systems will be guided by their quality, costs, and reimbursement models.

## Notes

Supplement 1. **eTable.** Input parameters of weekly symptom burden score

Supplement 2.
Data Sharing Statement
