# Supplementary material for: Cost-Effective Components of a Patient-Reported Symptom Monitoring System for Chemotherapy
Source: JAMA Netw Open. 2025 Nov 10;8(11):e2542289. doi: 10.1001/jamanetworkopen.2025.42289 (PMC12603855; doi:10.1001/jamanetworkopen.2025.42289)
Supplement: Supplement 1. — eTable. Input parameters of weekly symptom burden score [file jamanetwopen-e2542289-s001.pdf]

# Supplemental Online Content

Mooney K, Yoo M, Sloss ES, et al. Cost-effective components of a patient-reported symptom monitoring system for chemotherapy. *JAMA Netw Open*. 2025;8(11):e2542289. doi:10.1001/jamanetworkopen.2025.42289

**eTable.** Input parameters of weekly symptom burden score

This supplemental material has been provided by the authors to give readers additional information about their work.

© 2025 Mooney K et al. *JAMA Network Open*.

**eTable 1.** Input parameters of weekly symptom burden score

| Week | Group 1 SCC n = 143 | Group 2 SCC + AT n = 144 | Group 3 NP n = 148 | Group 4 NP + DSS n = 155 | Group 5 SCH SCC+NP+DSS n = 167 |
|---|---|---|---|---|---|
| 0 | 3.42 | 3.42 | 3.42 | 3.42 | 3.42 |
| 1 | 3.49 | 3.69 | 3.67 | 3.68 | 3.39 |
| 2 | 3.54 | 3.95 | 3.90 | 3.92 | 3.35 |
| 3 | 3.59 | 4.18 | 4.11 | 4.15 | 3.32 |
| 4 | 3.64 | 4.39 | 4.30 | 4.37 | 3.28 |
| 5 | 3.67 | 4.58 | 4.47 | 4.58 | 3.25 |
| 6 | 3.70 | 4.75 | 4.63 | 4.78 | 3.22 |
| 7 | 3.73 | 4.90 | 4.77 | 4.96 | 3.18 |
| 8 | 3.74 | 5.03 | 4.89 | 5.13 | 3.15 |
| 9 | 3.75 | 5.14 | 4.99 | 5.30 | 3.12 |
| 10 | 3.76 | 5.23 | 5.08 | 5.45 | 3.10 |
| 11 | 3.75 | 5.30 | 5.15 | 5.59 | 3.07 |
| 12 | 3.74 | 5.35 | 5.20 | 5.72 | 3.04 |
| 13 | 3.73 | 5.38 | 5.23 | 5.83 | 3.01 |
| 14 | 3.70 | 5.39 | 5.25 | 5.94 | 2.99 |
| 15 | 3.67 | 5.39 | 5.25 | 6.03 | 2.97 |
| 16 | 3.64 | 5.36 | 5.23 | 6.11 | 2.94 |
| 17 | 3.59 | 5.31 | 5.19 | 6.18 | 2.92 |
| 18 | 3.54 | 5.24 | 5.14 | 6.24 | 2.90 |
| 19 | 3.49 | 5.15 | 5.06 | 6.29 | 2.88 |
| 20 | 3.42 | 5.04 | 4.97 | 6.32 | 2.86 |
| 21 | 3.35 | 4.91 | 4.87 | 6.35 | 2.84 |
| 22 | 3.28 | 4.76 | 4.74 | 6.36 | 2.82 |
| 23 | 3.20 | 4.59 | 4.60 | 6.36 | 2.80 |
| 24 | 3.11 | 4.40 | 4.44 | 6.35 | 2.79 |
| 25 | 3.01 | 4.19 | 4.26 | 6.33 | 2.77 |
| 26 | 2.91 | 3.96 | 4.06 | 6.30 | 2.76 |

© 2025 Mooney K et al. *JAMA Network Open*.
