# Supplementary material for: Cost-Effective Components of a Patient-Reported Symptom Monitoring System for Chemotherapy
Source: JAMA Netw Open. 2025 Nov 10;8(11):e2542289. doi: 10.1001/jamanetworkopen.2025.42289 (PMC12603855; doi:10.1001/jamanetworkopen.2025.42289)
Supplement: Supplement 2. — Data Sharing Statement [file jamanetwopen-e2542289-s002.pdf]

Data Sharing Statement

Mooney. Cost-Effective Components of a Patient-Reported Symptom Monitoring System for Chemotherapy. *JAMA Netw Open*. Published November 07, 2025.
doi:10.1001/jamanetworkopen.2025.42289

Data
Data available: Yes
Data types: Deidentified participant data, Data dictionary
How to access data: The data that support the findings of this study are available from the corresponding author (Kathleen Mooney, kathi.mooney@nurs.utah.edu) upon reasonable request.
When available: With publication

Supporting Documents
Document types: None

Additional Information
Who can access the data: Researchers whose proposed use of the data has been approved
Types of analyses: For a specified purpose
Mechanisms of data availability: With a signed data access agreement
